# Investigating public awareness, prevailing attitudes and perceptions towards domestic violence and abuse in the United Kingdom: a qualitative study

**DOI:** 10.1186/s12889-022-14426-9

**Published:** 2022-11-08

**Authors:** Vasumathy Sivarajasingam, Iman Webber, Eva Riboli-Sasco, Aos Alaa, Austen El-Osta

**Affiliations:** 1 Department of Primary Care & Public Health, School of Public Health, Imperial College London, Charing Cross Hospital, 323 Reynolds Building, St Dunstan’s Road, London, W6 8RF England; 2 GP Partner, Hillview Surgery, Perivale, England

**Keywords:** Domestic violence, Abuse, Community setting, COVID-19, Rapid screening tools, Raising awareness, Interventions

## Abstract

**Background:**

Reported cases of Domestic Violence and Abuse (DVA) have increased since the advent of the COVID-19 pandemic and ensuing lockdowns. Understanding the general public’s view about DVA is vital, as it would help develop targeted interventions and effective public policies to tackle this rising problem in society. Our qualitative study investigated the public awareness, attitudes and perceptions towards DVA, and explored mechanisms to tackle DVA in the community setting in the UK.

**Methods:**

The research team conducted personal interviews with 29 community dwelling adults who responded to study invitations and adverts on social media. We used a topic guide to ensure consistency across the interviews, which were audio-recorded, transcribed and analysed thematically to detect emergent themes concerning DVA.

**Results:**

All respondents were aware of the concept of abuse. Thirty-eight percent declared either having experienced DVA directly or that they knew someone close to being abused. More than half of the respondents were not aware of existing DVA supportive services in the UK. Overarching themes generated from the contextual analysis included contributing factors for DVA, challenges and barriers facing victims and proposals for future interventions.

**Conclusions:**

Community dwelling adults have a good understanding of the impacts of DVA, but many fail to recognise specific instances or events in their daily lives contributing to DVA. Raising public awareness, particularly in children through the school curriculum, highlighting existing support services and introducing the routine use of short screening tools for DVA in health and social care settings can increase awareness, early identification and signposting to effective interventions. Sustained, multi-level community facing interventions are recommended to reduce stigma and fear associated with DVA.

**Supplementary Information:**

The online version contains supplementary material available at 10.1186/s12889-022-14426-9.

## Background

Domestic violence and abuse (DVA) is one of society’s ‘wicked’ problems and violation of human rights. It is a common public health concern and continues to be an issue among families, impacting both the mental and physical health and wellbeing of all who are exposed including perpetrators, victims and children who witness abuse [1]. Crime Survey for England and Wales (CSEW) for the year ending March 2020 estimated that 5.5% of adults aged 16 to 74 years (2.3 million) experienced DVA in the last year [2]. A study by the Home Office estimated the cost associated with DVA for the year ending on 31st of March 2017 equated to approximately £66 billion [3].

DVA can affect anyone, regardless of age, ethnicity, gender, sexuality, class, lifestyle or geographic location [4, 5]. Often people suffering from DVA have unnecessary investigations and medications to address a variety of physical and/or mental health symptoms, including chronic pain, and are frequent attenders to the healthcare service [6].

An individual’s early life experiences is important to their health throughout their life course [7]. Those who had adverse childhood experiences (during childhood or adolescence) tend to have more physical and mental health problems as adults than those who did not, and ultimately greater premature mortality [7]. One in seven children and young people under the age of 18 will have lived with DVA at some point in their childhood [8]. Witnessing DVA can lead children to develop an array of age-dependent negative effects including short and long term cognitive, behavioural and emotional effects, in addition to direct physical consequences including injuries and even death [9, 10]. Children exposed to DVA are more likely to either experience or perpetrate DVA as adults [9].

The main reasons why victims face barriers to seeking help include stigma, shame, fear of reprisal, financial implications and perceptions that support may not be available or adequate [5]. This is compounded further as some victims do not realise they are experiencing abuse, so there are major concerns that DVA cases in general are especially unrecorded and unreported [11]. Victims of DVA are often at different stages in their readiness to disclose their experiences of abuse and may minimise it. Raising awareness of the negative consequences of DVA in the society, would not only help survivors of DVA to openly discuss and encourage receiving support but will also support victims who are suffering in silence to recognise and acknowledge the abuse they are experiencing. The World Health Organization (WHO) encourages the health sector to play a crucial role in prevention [12]. Raising awareness about DVA in the community setting can also translate to people involved in violence becoming more aware of the help and support they can rely on to tackle this rising problem in society.

Following the advent of the COVID-19 pandemic, outreach to the UK National Domestic Abuse Helpline website increased by 700% in the second quarter of 2020 compared with the first quarter [13]. Studies have shown moderate to strong increase in DVA incidents following the COVID-19 related restrictions (i.e., stay at home orders, lockdown orders) coupled with financial difficulties, closures of key community resources, social isolation and victims being forced to stay indoors with the perpetrators [14–16]. Stay-at-home mandates have also amplified pre-existing mental health conditions and psychosomatic distress reactions [14]. As the usual channels of support were jeopardised by lockdown restrictions and social distancing, those suffering abuse needed to find alternative means of support and safety with a particular focus on evolving digital and technology based DVA mitigation strategies [14]. Survivor voices, rights and perspectives is a priority in the design of trauma-informed digital intervention [14].

The cost of DVA, in both human and economic terms, is so significant that even marginally effective interventions are cost-effective [17]. Understanding the general public’s knowledge, attitudes and perceptions about DVA is vital, as an awareness about the prevailing societal perceptions about this complex public problem can help guide the development of targeted interventions and the formulation of effective public policies. The literature on community attitudes towards DVA from the UK is limited. This study aims to investigate people’s awareness, attitudes and perceptions of contributing factors and barriers to tackling DVA and explores recommendations for interventions to tackle DVA in the contemporary setting.

## Methods

We utilised a constructivist qualitative methodological approach involving a semi-structured interview framework to address the aims of the study. A full description of the method is included in Supplementary File 1.

As our research question about DVA is sensitive and exploratory in nature, we chose a qualitative semi-structured interview guide, combining elements of structured and unstructured interviews. The open-ended nature of the interview guide offers comparable, reliable data with the flexibility to ask follow-up questions, contributing to the objectivity and trustworthiness of studies and makes the results more plausible [18]. We used a purposive sampling technique and potentially eligible participants were contacted via email through community groups in the London Boroughs of Hammersmith & Fulham, Brent and Harrow catchment area, and using adverts on social media. This included all members of the community, provided they fit within the inclusion criteria.

Potentially eligible participants were given a Participant Information Sheet which provided comprehensive information on the study’s purpose, aims, the reason why they were chosen, possible benefits, disadvantages, risks of taking part in the study and the interview process, which included information on online access and internet browsing history ensuring confidentiality and anonymity were maintained. They were also informed about the interviewer (their designation and level of experience of conducing qualitative research), the length of the interview, where the data was stored and for how long. Participants were reminded of the voluntary nature of this study and were given enough time to decide if they wanted to participate. They were informed that they could withdraw from the study at any point, without giving a reason leading up to, or during the interview; advised not to answer questions they were uncomfortable with, without giving any explanations. In addition, the participants were signposted to some useful links including DVA helplines and support services for advice and support should they need at any stage.

Due to the confidential and sensitive nature of this topic, the interview was scheduled following the participant’s written consent at a convenient time, provided they were alone without anyone listening in or potential intrusion from anyone. This was confirmed by the verbal consent of the participant before starting the in-depth semi-structured interview. In case a situation arose where someone entered the room unexpectedly or there was a distraction, the interview would be terminated for the safety of the person. The participant also had the option to leave the interview at any point without an explanation.

Semi-structured one-to-one personal interviews with participants who responded to the study invitation were conducted via telephone, Microsoft Teams or face-to-face in line with social distancing guidelines between 28 July and 8 September 2021. The duration of the interviews varied between 15 and 45 min, with the average being 35 min.

Contextual data gleaned from personal interviews were analysed to detect main and emergent themes concerning DVA. The interviews sought to (1) understand public awareness about the prevalence of DVA in the community since the COVID-19 pandemic, (2) identify people’s views and experiences of DVA, including familiarity with contributing factors, (3) explore the perceived barriers and challenges faced by victims of DVA, and (4) identify people’s opinions of recommendations of interventions to support victims and prevent abuse. The research team developed the interview topic guide after defining the research objectives and reviewing relevant literatures, provided as Supplementary File 2. The Consolidated Criteria for Reporting Qualitative Research (COREQ) was used to guide reporting (Supplementary File 3). The study received a favourable opinion from Imperial College Research Ethics Committee who approved the study (ICREC #21IC6721).

### Data analysis

The research team comprised of one female general practitioner who conducted the personal interviews with support from co-authors, including one clinical academic, two mixed methods researchers and one statistician. All members of the study team supported the development of the study protocol, study design, data collection and data analysis, and were experienced in conducting qualitative research.

As this is an exploratory study, interview transcripts were coded using a process of open coding (Supplementary File 4) by VS in discussion with the research team, followed by the development and clustering of themes in an interpretive process. The basic codes were elaborated into a framework that was continuously refined to reflect all the interviews (Supplementary File 5). The emergent themes were checked against the interview guide and study objectives, resulting in the development of a set of major themes. Co-authors of the study verified the emerging themes and contents. We used the socio-ecological model as a framework [19] to illustrate how the proposed intervention strategies could be used to tackle DVA at the individual, relationship, community and societal levels.

## Results

### Study participants

We interviewed 29 participants who had varied backgrounds and experiences (Table 1)**,** with an age ranging between 18 and 72 years. The mean age of respondents was 42 years (Standard Deviation 15.02). More than two-thirds (69%) of respondents were female. The vast majority (79.3%) had a university degree or higher and the remaining finished high school. The overarching majority were employed either full time (82.7%) or part time (10.3%). Our study findings showed that participants’ gender, age and ethnicity did not have an impact on their attitudes or perceptions towards DVA.

**Table 1:** Participant characteristics

	**N**	**Percentage**
**Age**		
18–29	8	27.6%
30–39	6	20.7%
40–49	5	17.2%
50–59	6	20.7%
60–69	3	10.3%
70–79	1	3.4%
**Gender**		
Female	20	69.0%
Male	9	31.0%
**Ethnicity as per 2021 Census of England & Wales **[20]		
White	16	55.2%
Mixed/Multiple ethnic groups	0	0.0%
Asian/Asian-British	10	34.5%
British Black/African/Caribbean	1	3.4%
Other (Arab or any other ethnic group)	2	6.9%
**Education**		
Did not finish high school	0	0.0%
High school	6	20.7%
University degree or higher	23	79.3%
**Employment**		
Employed full time	24	82.7%
Employed part time	3	10.3%
Unemployed	0	0.0%
Furloughed	0	0.0%
Retired	0	0.0%
Student	1	3.4%
Unable to work	1	3.4%

Eleven respondents (37.9%) had either personally experienced DVA (13.8%) or were aware of someone close being abused. Three respondents admitted having experienced some form of psychological abuse, whereas all felt that emotional and psychological abuses could be easily disguised and overlooked, leaving deep-rooted mental wounds, taking a long time to recover and having enormous bearing on future relationships, as it ‘continuously chips away’ at their mental state and belittles them.

‘… the physical abuse we see is only the tip of the iceberg’ (M 19)

All participants perceived an increase in the prevalence of DVA since the advent of the COVID-19 pandemic. The majority felt this was due to people not being able to ‘let out’ their frustrations due to forced isolation, reduced social contact, anxieties about the future and the closure of sporting and entertainment venues which could have otherwise relieved stress and diminished occurrences of abuse.

Three overarching themes were generated from the contextual analysis: (1) contributing factors to DVA, (2) challenges facing the victims, and (3) proposals for future interventions (Table 2). A fuller description of the themes and resultant framework are included in the Supplementary File 5. From the respondents' feedback, we identified three categories of proposed interventions to support victims and perpetrators of DVA: (1) raising public awareness, (2) enhancing provision of support services and, (3) education and training throughout the life course.

**Table 2:** Overarching themes

Categories	Subcategories	Thematic framework
**Contributing Factors**	• Victims • Perpetrators	1. Experience 2. Personality types 3. Feelings & Beliefs 4. Sociocultural aspects 5. Religious beliefs 6. Circumstances 7. Finance 8. Children & other dependents 9. Substance abuse 10. Vulnerable/ ill-health
**Challenges Facing Victims**	• Barriers to seeking support • Societal beliefs • Individual attitudes & behaviours • Prevailing legal /policy framework • Practicalities & logistics • Support mechanisms	11. Social conditioning 12. Cultural/religious constraints 13. Circumstances 14. Perceptions 15. Fear 16. Physical & mental vulnerability 17. Disability 18. Lack of education & training 19. Lack of awareness 20. Lack of accessible support 21. Limited communication 22. Children & other dependents 23. Lack of tools to identify ‘hidden abuse’
**Proposed Interventions**	• Raising Awareness • Education & Training • Reducing stigma • Routine signposting to screening tools for DVA • Guidance & mobilization of support services	24. Improve public awareness 25. Improve self-awareness & holistic wellbeing 26. Promote social support & break social silence 27. Raise awareness in school curriculum 28. Workforce training 29. Support from family & friends 30. Housing/shelter 31. Financial assistance 32. Legal support 33. Counselling 34. Improved communication 35. Accessible support services 36. Screening/ better tools to help identify ‘hidden abuse’

#### Contributing factors to DVA

##### Reasons why victims continue to experience DVA

Cultural and religious beliefs were stated as central contributing factors, such that victims were not initially aware that they are experiencing the abuse.

‘It’s what happens in every household – so it’s normal’ (F5)

A few participants identified that in certain cultures, male dominance hierarchy is a widely accepted ‘social norm’, which supported violence as a means of conflict resolution. These gender norms have an impact on women’s roles and access to resources and influence the extent of personal involvement in decision-making at all levels.

*'Many people in such cultures, become complacent and become submissive, and follow the culture the society has prescribed to them; so, they internalise this and it becomes normal to be abused’ (F10)*

Respondents lamented that victims often suffered in silence and often did not come forward because they feared a lack of anonymity, and of the unknown, and this was usually coupled to a lack of trust in the system.

‘I feel I will be looked down by others—especially as a male victim, [because] as a man you shouldn’t be abused’ (F3)

Participants were mindful that anyone could be exposed to abuse. Poverty was linked with DVA as both a cause and a consequence. Conversely, one participant stated that DVA may be syndemic with affluence and therefore also present in wealthy or ‘powerful’ families, but this also tends to be easily ignored or remain unquestioned.

##### Reasons why perpetrators abuse victims

Participants acknowledged that any kind of abuse was unacceptable and unjustifiable but indicated that perpetrators are compelled to commit abuse due to certain personality traits such as a lack of self-confidence, and/or mental illnesses. Others suggested that the abuser’s motivations stemmed from a desire to gain control, or from a lack of marital/relationship satisfaction or financial worries, especially with job loss and associated substance misuse. Thus, whereas stress at the workplace or home environment may cause an inflammatory response in a relationship, and subsequently this may translate to DVA, the ‘perpetrator as victim’ standpoint was not justifiable. A minority of participants voiced that some abusers are ignorant of their actions, and that such demeanour is established as ‘the norm’ by the children and young adults, who in turn may carry on the behaviour.

#### Challenges facing the victims

Sociocultural and religious constraints played a substantial part in victims facing challenges. Other factors included stigma, fear of repercussion, shame, embarrassment, not wanting to be judged, considering DVA as a private matter, lack of support from family/friends, fear of loneliness, lack of alternative housing or lack of job skills. Victims may feel guilty, give excuses, blame themselves for the abuse, experience feelings of powerlessness or feeling that they have no choice or being ‘forced’ to stay for the sake of their children who ‘need a father’. Some feel safer and that it is more convenient to stay with the perpetrator because the abuser provides the monetary assistance, financial security, or the necessary amenities for the couple to care for their dependents. Participants also expressed the victim’s unawareness of undergoing abuse or accessibility of support services as a considerable reason for not seeking support.

#### Proposals for future interventions

Respondents considered what type of interventions or initiatives could be implemented to support victims and perpetrators. Whereas the majority acknowledged that perpetrators could themselves be considered as victims of previous DVA, they did not feel that perpetrators are likely to be remorseful for treating their ‘loved ones’ improperly. Hence, they emphasized the importance of interventions to support both parties, with a specific emphasis on children.

- (1) Raising public awareness of DVA and accessible support
  Most respondents attributed public awareness to news and social media. Posters displayed in public toilets, including waiting rooms in GP surgeries were considered beneficial in raising awareness, whereas campaigns targeting specific at-risk groups were welcomed. Those participants who experienced DVA had supportive relatives, and hence felt better able to ‘cope and get on with life’.
  ‘Raising public awareness and self-awareness is the key in terms of what should be and shouldn’t be acceptable’ (M19)
  More than half (52%) of respondents interviewed reported not knowing what services, helplines or resources they could rely on should they experience DVA. Those who knew about the supportive services or helplines included survivors with previous experiences with DVA and healthcare professionals (HCPs) who had special interests in DVA or supported people experiencing abuse. The participants highlighted a need for more publicity and awareness of support services like safe spaces, helplines, organisations and charities to be more visible and accessible to the public. Participants felt that such information should be advertised widely in various languages to address the UK’s multi-ethnic population, including the use of visual infographics to communicate salient points.
- (2) Enhancing provision of supportive services
  ‘No point being aware of domestic abuse, people should be clear on the pathway’ (F5)
  Alternative accommodation such as ‘emergency hotels or safe spaces’ was recommended by all as a means of immediate support for victims. Anonymous helplines and online chats at all hours of the day were welcomed. Few mentioned having access to free counselling services, and a ‘safe social house’ with psychologists and social workers as a step forward in tackling abuse. Financial assistance, provision of childcare and housing were advocated as an optimistic step towards motivating victims to seek support. Mental health issues were recognised as a central contributing factor for both victims and perpetrators. Investing resources in this avenue were advocated by many to unravel the inherent issues relating to DVA. All felt that familiarity with the pathways, having quick access and speedy response from support services would encourage victims to pursue help.
- (3) Education and training
  All participants stressed the importance of preventive measures such as training on measures to prevent DVA throughout the life course, which should be implemented in the school setting and across the general population. Such course of actions would help support early identification of DVA, disclosure of abuse and support perpetrators as well as survivors of abuse.

### Schools

All participants emphasized that schools could be an ideal place to educate children about DVA and what support mechanisms are in place to tackle this rising problem of society, especially if education is tailored appropriately to the age group (such as interactive play for primary school children). In the UK, the school curriculum includes teaching children about healthy relationships, though often not prioritised by schools. Teachers should also have better training and attentiveness to identify and support children who are exposed to DVA. A majority felt it should be the schools’ responsibility rather than the parents to teach children about DVA as it would help provide more consistent guidance and advice. This approach was encouraged as it unquestionably helps to circumvent differences in social and cultural perspectives at home and further cultivates better awareness of DVA in the long-term.

*‘Children learn their behaviour by watching their parents; we need an external* *and internal source to educate children; hence a united learning that recognises the unacceptable behaviour’ (F17)*

### General population

Participants felt no single or ‘one-size fits all’ strategy could combat the myriad of reasons contributing to the problem. Widespread publicity would capture the attention of the general population.

Being vigilant and responsible in supporting the victims in the community should be the prime aim in dealing with such a diverse, sensitive and preventable problem. Theatre and dance companies could collaborate in a creative way reaching out to people, raising social consciousness around DVA and supporting survivors to express their experience. This could create a safe space for victims to reveal the challenges they have faced and celebrate their endurance with the audience.

### Early identification

Routine touchpoints with HCPs in the NHS and social care settings were considered ideal opportunities to identify abuse, and this assumption was also confirmed by the HCPs interviewed. Most respondents recognised screening as an effective means for early identification of victims. However, a few (mostly GPs) highlighted time constraints as the main barrier for the widescale implementation of DVA screening during routine consultations.

‘Victims will come forward if people are willing to listen to their stories’ (F3)

### Disclosure of abuse and support

The emphasis was primarily around unlearning the learnt behaviour, for both the victims and perpetrators.

### Victims

Education on attitudes and behaviour to avoid re-victimisation was highlighted by a majority of participants.

‘We need to identify the teachable moments when victims are receptive to receiving information; this is the time to share facts’ (M19)

Ensuring that anonymity is maintained was identified by many as a way of improving victims seeking help, potentially giving a new identity. Participants highlighted those children who witness abuse including overhearing or observing the abuse, or those who directly experience the abuse, can also be considered as being exposed to DVA. There was a clarion call from respondents that child victims need a supportive parent/adult to get through this ordeal. As abuse is a learned behaviour, focus should be on children to stop evolving victims or perpetrators.

### Perpetrators

Participants established that abusers will not willingly talk about their conduct, even if they are aware of the distress they are causing their victims. Hence, having helplines and receiving assistance without being criminalised would be a productive step.

The majority felt that perpetrators are victims themselves and might be unaware of their behaviour; this could be related to their upbringing in a violent environment. Few felt a level of empathy should be given as most abuses come from unresolved trauma and if appropriate therapy was given to the abuser, they would gain insights to reshape their morals and live more fulfilling lives.

Finding the trigger factor might be a helpful way to support perpetrators. They should be offered counselling and/or rehabilitation. The majority felt that sending the abuser to prison may not solve the matter in terms of behavioural change. Education was emphasized to be vital in stopping repeat offences or being victims themselves. However, a few participants felt strongly this should be in addition to the prison sentence as this will deter others from committing such crimes.

## Discussion

To the authors' knowledge, this is the first study that explored public attitudes and perceptions concerning DVA, and the use of a short screening tool during routine touch points in community, health and social care settings in the UK.

Our findings highlighted that whereas all respondents were aware of the concept of abuse, people’s awareness of DVA may have increased somewhat due to the broad publicity it has received during the first national lockdown following the advent of COVID-19. This is supported by the world’s longest-running survey, The National Community Attitudes toward Violence against Women Survey, reporting the majority of Australians’ levels of awareness towards violence against women have generally risen [21]. Indeed, whereas CSEW estimated a DVA prevalence rate of approximately 5 in 100 adults in the year ending March 2021 [22], 18% of all the crimes recorded by the police during that same period were due to DVA incidents, equating to a rise of 6% from the previous year [22].

Just over a third of our study respondents reported having been exposed to or experienced some form of abuse. The main reason for non-disclosure was emphasised as the lack of public recognition attributed to normalisation or social silence with its associated fear, stigma and ‘lack of trust in the system’ at local and national levels. Our findings corroborate a recent UK study on South Asian women which revealed the numerous ways in which denial of citizenship continues even long after the end of the abusive relationship, and the efforts needed to regain a sense of identity, belongingness and membership within their intimate, family and community lives [23].

That only half of the participants in the study were aware of existing support services in the UK was consistent with previously published research confirming that DVA cases are vastly unreported [11]. Feedback from respondents also echoed the findings of a recent NHS survey which showed that two in five people were unsure or did not know where to get help after being abused, and more than half of people did not ultimately seek help following their experiences of abuse [24]. DVA organisations including The Survivors Trust and politicians are also attentive that many victims and survivors are unaware of the specialist support available to them and how to access it [24]. As one respondent states, this shows that the abuse recorded is only the ‘tip of the iceberg’ in the UK, largely because most victims may not access support for what some individuals deem to be a ‘private matter’.

Many of the interventions proposed by our study participants have already been implemented in the UK, reinforcing the significance of simultaneously raising public awareness and improving the visibility of accessible support mechanisms for both victims and perpetrators. A new campaign launched in February 2022, coinciding with the first day of UK’s Sexual Abuse and Sexual Violence Awareness Week, aims to raise awareness of the centres and support available in England to those experiencing sexual assault, abuse or rape, including those not knowing who or where to turn to [24]. This is an important step to help raise public awareness of DVA and is the largest such campaign in the UK since the advent of COVID-19.

Consistent with our findings, a recent study of DVA victims in rural communities in Southern Ontario recommended outreach programs and campaigns in supporting public and professional education [25]. Another study conducted on Chinese university students suggested that education plays a powerful factor in influencing perceptions and attitudes concerning DVA compared to factors such as gender, residence and age [26].

### Prevention strategies using the socio-ecological model

The Centers for Disease Control and Prevention uses a four-level social-ecological model to analyse the interplay between individual, relationship, community and societal factors, to better understand violence and the effects of potential prevention strategies [27]. Multi-level interventions are needed to sustain prevention efforts over time and achieve population-level impact. In our analysis, we used the socio-ecological model as a framework [19] to illustrate how the respondents’ recommended prevention strategies could be used to tackle DVA in our society (Table 3). This approach sheds light on how dynamic interactions across multiple domains ranging from individual risk factors to broad social factors could contribute towards the risk and protective elements for DVA [9]. It also highlights how preventive interventions can be developed to work across four distinct levels: individual, relationship, community and societal.

**Table 3:** Socio-ecologic grouping of proposed interventions to tackle DVA in the community setting

Level	Example intervention	Setting
Individual (Micro level)	• Personal empowerment • Self-referral/ Online referral • Counselling (medical, psychological, legal) • Coping strategies when abused	Home setting
Relationship (Meso level)	• Friendships/ community support • Individual social responsibility in the community • Mentoring • Teaching/ skill-building programs • Rehabilitation	Education, workplace & community settings
Community (Meso level)	• Use of screening tools for early identification • Increased visibility of existing support services • School curriculum • Education and training initiatives • Workplace support • Online resources. Free helplines • Awareness raising campaigns • Police protection orders & prosecution service	
Societal (Macro level)	• Promoting social norms • Policies and legal framework to support victims • Funding, charities, supportive services including safe spaces • Advocacy	Social milieu, society & culture

As there can be no ‘one size fits all’ approach to tackling DVA, the socio-ecological lens reinforces the importance of developing a comprehensive approach in which actions at each level of the social ecology synergise with interventions implemented at other levels [19]. Similar approaches have been utilised in other DVA studies proposing potential prevention strategies, practice and policy implications [28]. Multi-level programs are most effective in changing behaviour, but there is consensus that any such interventions need to be funded and sustained for several years to make any real impact on the actual cases of DVA.

At the individual (micro) level, raising awareness educates and influences people to change their attitudes, behaviours and beliefs, thus helping to shift public opinion and sway the political will of decision-makers [29]. At the relationship and community (meso and macro) level, public education campaigns focussing on the individual’s social responsibility in the community may also help change some of the prevailing and largely unhelpful societal attitudes towards DVA such as victim-blaming, silence, tolerance, stigma and inhibition, and could make a substantial contribution to preventing abuse [30].

It is widely acknowledged that social support leads to positive mental health outcomes, improved quality of life and more willingness to seek formal support and physical safety [17, 31]. Having open dialogues about the detrimental health consequences of abuse in society, coupled with more awareness about appropriate referral pathways and linkage with local support services, including helplines might motivate survivors to pursue support. This mobilization could also prompt support networks to encourage those who are ignorant or inhibited due to social silence to come forward. By breaking this ‘deafening code of silence’ and reducing social tolerance and inhibition, individuals, health systems and society can take the necessary steps towards the challenge of ‘melting the iceberg’ of DVA. This could also help raise awareness among perpetrators who may become more accepting of receiving support [32].

### Routine use of screening tools

Assessment tools and guidelines are available to help promote the recognition of and outline the support available to people experiencing DVA [33, 34]. Studies have shown that routine DVA screening improves victim identification in healthcare settings [32], playing a key mechanism in reaching and supporting the victims, particularly those who may not engage with other services. Currently, the National Institute for Health and Care Excellence (NICE) in England does not recommend the use of validated tools for routine screening of DVA [35]. The US is one of the few countries with a policy of screening for DVA, but some of the evidence for screening in healthcare settings is contraindicatory [36]. For example, there are no head-to-head trials of screening versus clinical enquiry, and we do not know which is more effective. Nevertheless, screening programmes are not all that different from targeted inquiry approaches [36]. Thus, whereas our study findings also highlight that routine screening can be an effective means for early identification of victims, other studies showed that many doctors do not implement screening because of time constraints, lack of training or discomfort with asking about abuse [36] which was reiterated by our study HCPs.

Any contact between the patient and the healthcare system offers a window of opportunity to diagnose abuse or neglect [37]. Routine enquiry of DVA, even when there are no obvious indicators of abuse could assist in early identification, raising public awareness and tackling the issue before it escalates. Our study findings suggest that routine touchpoints with HCPs in the NHS and social care settings to be an ideal opportunity to identify those experiencing abuse. Asking all patients standard questions can help with this and at the same time highlight to victims that they are not alone in their experiences. Additionally, this may even prompt perpetrators of abuse to recognise and seek support. Examples of brief screening tools for DVA include the Woman Abuse Screening Tool (WAST), WAST-Short, and Hurt-Insult-Threaten-Scream (HITS) tool [38–40]. In this regard, HCPs have a crucial role in tackling DVA, especially when utilising rapid assessment tools to identify abuse, when signposting to suitable services or when helping promote the recognition of and outline the support available to victims [34, 41].

### Rationale for using routine touchpoints with health and social care to screen for DVA

The working age (16–65 years) population of the UK consists of 34.4 million individuals [42], equating to 51.3% of the total UK population which was 67 million in 2020 [43]. The NHS workforce alone comprises of 1.4 million individuals [44], whereas the social care workforce is 1.54 million [45]. Combined, the health and social care (H&SC) workforce comprises of 2.94 million individuals, equating to 8.6% of the total working population. The UK’s large H&SC workforce routinely engages with the vast majority of the total UK population on an annual basis (i.e., during touchpoints with a HCP, GP, specialists in secondary care, or allied health professionals for reablement or social care), which makes it ideally suited to raise awareness and screen for DVA using short, validated tools. The provision of on-going training and support to the H&SC workforce is necessary to improve the professionals’ confidence in the identification, guidance, and referral of victims to the existing DVA support services. It is crucial to increase access to effective screening tools in order to make it easier for HCPs to assist victims in disclosing information about DVA so that the root cause could be addressed. As screening plays a central role in the early identification of DVA, particularly unreported and easily hidden abuse (e.g., psychological, financial, coercive, and controlling behaviour), we recommend that the routine use of validated screening tools for DVA be considered by community and NHS primary care services to promote the timely identification of victims for signposting and referral to appropriate support services.

### Education and training

Rapid screening for DVA during routine H&SC touchpoints should be supported by structured education and training in the school setting. Most respondents proposed that a DVA awareness exercise should ideally be integrated into the school curriculum, and to feature as part of the education workforce induction and mandatory training, but this is unlikely to happen at scale without the support from policymakers. Entertainment venues also have a momentous role in educating the community, via creative interaction and by providing a safe place for victims to seek help.

‘Everyone has a role in ending domestic abuse; together we can create a society that no longer tolerates abuse’ (F20)

Serious case review findings show that death or serious harm might have been prevented if H&SC professionals had acted upon their concerns or sought more information [46]. This makes the case for more pervasive use of short DVA screening tools (e.g., WAST-Short), and those multiple strategies to tackle DVA throughout the life course are needed with consistent funding and support from policymakers.

In summary, raising public awareness, enhanced education and training of people from all walks of life and throughout the life course coupled to the routine utilisation of screening tools for early identification of DVA can help tackle this ‘wicked’ problem of society. Collaborative efforts from every layer of society and organisations including schools, communities, workplaces, healthcare settings, law enforcement bodies and politicians are required to keep DVA ‘relevant’ via public awareness campaigns to affect a positive change in social attitudes and the visibility of support services.

### Study limitations

Because our study sample was small and from a localised area, the findings of our study are not necessarily representative of the UK population. Nevertheless, useful insights into personal experiences were provided by this small cross-section of respondents. In qualitative studies, the pragmatic sample size is often considered sufficient when saturation of themes is nearly accomplished [47]. We feel our data was sufficient in this respect. We acknowledge that additional interviews may have resulted in the identification of other emergent themes, particularly with respect to considering the perspective of perpetrators and not just individuals who may have suffered abuse. Inevitably, the study sample included some selection bias [48], such that only those with an interest or who experienced DVA, or those who were in employment or highly educated participated in the interview, but the breadth of contextual data we explored was adequate given the sensitive nature of the topic. A larger study with a more diverse cross-section of British society is indicated, including data collection from the health and social care workforce, policy makers and commissioners of wellbeing support services.

## Conclusions

To our knowledge, this is the first study in the UK that explored public attitudes and perceptions concerning DVA in the community setting since the advent of COVID-19. Sustained, multi-level community-facing interventions need to be implemented and targeted at individuals from all walks of life and throughout the life course to change the social climate and break the code of silence on DVA and continue to bring this issue into the public light. If we are serious about tackling the issues of DVA with a view to improve prevention, NICE may consider recommending the routine use of a rapid screening tool for DVA which could be administered by health and social professionals during the millions of routine contacts per year. The health and social care workforce represent nearly 10% of the total UK working age population and may help with early identification and supporting of survivors of DVA. The use of rapid screening tools such as the WAST-Short during routine H&SC touchpoints, the inclusion of DVA education in educational settings, and the sustained funding of multi-level interventions can help introduce new values, thinking processes and relationship skills that are incompatible with abuse.

## Supplementary Information

**Additional file 1. **Methods. A full description of the method.

**Additional file 2. **Interview Guide - core and probe questions, asked in semi structured interviews that relate to participant’s DVA experiences. The research team developed the interview topic guide after defining the research objectives and reviewing relevant literatures

**Additional file 3. **COREQ checklist. COREQ was used to guide reporting.

**Additional file 4. **Preliminary codes and related illustrative quotes. A fuller description of the preliminary codes and quotations from individual participants.

**Additional file 5. **Preliminary codes, initial thematic framework and final categories and subcategories. A fuller description of the themes and resultant framework.

## Data Availability

The datasets analysed during the current study are not publicly available to protect the privacy of participants but are available from the corresponding author on reasonable request.
