# Supplementary material for: Investigating public awareness, prevailing attitudes and perceptions towards domestic violence and abuse in the United Kingdom: a qualitative study
Source: BMC Public Health. 2022 Nov 8;22:2042. doi: 10.1186/s12889-022-14426-9 (PMC9643966; doi:10.1186/s12889-022-14426-9)
Supplement: Supplementary file 1 — Additional file 1. Methods. A full description of the method. [file 12889_2022_14426_MOESM1_ESM.pdf]

Supplementary File 1

Methods

Design and settings

A constructivist qualitative methodological approach involving a semi-structured interview framework was used to address the aims of the study. We used a purposive sampling technique [1] and potentially eligible participants were contacted via email through community groups (mainly, but not exclusive to, the London Boroughs of Hammersmith & Fulham, Brent and Harrow catchment area), and using adverts on social media. This included all members of the community, provided they fit within the inclusion criteria.

We conducted semi-structured one-to-one personal interviews with participants who responded to the study invitation, via telephone, Microsoft Teams or face-to-face in line with social distancing guidelines. Contextual data gleaned from personal interviews were analysed to detect main and emergent themes concerning DVA. The exploratory interviews helped to assess the knowledge, attitude and perceptions about DVA in the general population.

Participant Recruitment

A purposive sampling approach was used to recruit the study participants which included individuals who were 18 years and over-living in the UK and who spoke English. Email invite introduced the study and provided details of how they can participate in interviews. Respondents who were under 18 years of age, non-English

speaking, unable to cooperate due to learning disability for example or with no access to personal computer or smartphone with internet connectivity were excluded from the study. Potentially eligible participants were given a Participant Information Sheet (PIS), which provided comprehensive information on the study's purpose, aims, the reason why they were chosen, possible benefits, disadvantages, risks of taking part in the study and the interview process, which included information on online access and internet browsing history ensuring confidentiality and anonymity was maintained. They were also told who the interviewer was, the length of time of the interview, where the data was stored and for how long. The participants were reminded of the voluntary nature of this study and were given enough time to decide if they wanted to take part in the study. The participants were informed that they could withdraw from the study at any point, without giving a reason leading up to, or during the interview; advised not to answer questions they were uncomfortable with, without giving any explanations. In addition, the participants were signposted to some useful links including DVA helplines and support services for advice and support should they need at any stage.

Interviews were conducted between 28 July and 8 September 2021. All participants signed the consent form prior to the interview, as per the study protocol which was developed prior to study commencement. Before the interview, the participants were made aware that should there be any concerns during the interview process, the interviewer will offer recommendations of receiving advice and direct them to DVA helplines and support services. However, on an occasion when the interviewer felt that there were concerns about the immediate safety of a person, actions would be

taken to protect the person concerned. All participants provided consent to the publication of their anonymized responses.

All responses were pseudo-anonymised to ensure confidentiality by assigning each respondent a unique study ID. Only the participants' demographic data (age in years, gender, ethnicity, education, employment, marital status and the first segment of postal code) were recorded.

The conversation was audio-recorded with consent, and the recording was destroyed after transcription. All data and interview transcripts were stored securely on an encrypted and secure institutional server, which can only be accessed using passwords adhering to Imperial College London policies and procedures. Participants were informed that any ad verbatim quotations that might be included to illustrate key themes would be anonymised.

Data Collection

One-off in-depth qualitative interviews were carried out by VS, a female GP and honorary clinical research fellow at Imperial College London who has published articles and received specialised training related to DVA. All interviews took place face-to-face or remotely through Microsoft Teams or telephone, with a total of 29 participants meeting the inclusion criteria. Relationship with the interviewees was not established prior to the study commencement. All interviews took place ensuring privacy, with no one else present apart from the participant and interviewer. Interviews were audio recorded and transcribed to collect the data. All interviewees completed the interview and answered all the questions without a break or

terminating the interview. Neither the interviews nor the transcripts were repeated or returned to participants. The interview process was terminated when no new information was forthcoming and data saturation was reached. In qualitative studies sample size is often considered enough when further interviews will not yield new responses [2, 3]. Saturation of themes was realised at the 27th interview; however, two further interviews were conducted to confirm the saturation. The duration of the interviews varied between 15 and 45 minutes, with the average being 35 minutes. Field notes were written immediately after each interview to ensure the flow of thoughts without interruptions.

The interview questions were open, semi-structured and designed to explore participant's experiences, attitudes and views in more depth (Supplementary File 2). The questions were first discussed with the research team and an interview guide was developed to provide structure and focus, which was piloted during the first three interviews conducted by VS (face validity) and refined further based on an in-depth literature review [2] . Probing questions were asked and participants were encouraged to express additional opinions and comments. Specifically, our objectives were to:

1. Understand public awareness about the prevalence of DVA in the community since the COVID-19 pandemic
2. Identify people's views and experiences of DVA, including familiarity with contributing factors
3. Explore the perceived barriers and challenges faced by victims of DVA
4. Identify people's opinions of recommendations of interventions to support and prevent abuse

Data Analysis

The research team consisted of the first author (VS) who undertook the interviews, with support from co-authors (n=3) who were involved in developing the study protocol, study design, data collection and data analysis. Interviews were listened to, and transcripts read several times to capture all the information provided by the participants. Notes were made under each of the four objectives including ad verbatim quotations using the information provided by each participant. Interview notes were read thoroughly and thematically analysed for their contents (familiarization; generating initial codes; searching for themes; reviewing themes; defining and naming themes). Initially, each transcript was coded using a process of open coding [See Supplementary File 4] by VS in discussion with the co-investigators (n=3), followed by the development and clustering of themes in an interpretive process. The basic codes were elaborated into a framework that was continuously refined to reflect all the interviews (Supplementary File 5). The study team did not discuss findings with participants but were keen to share publications with anyone who expressed interest.

In this study, credibility, transferability, dependability, conformability, and a reflexive approach to the inquiry and analysis were used to establish rigor and trustworthiness [4]. To ensure this, we provided a comprehensive description of the study methodology, and the findings were supported by verbatim responses from the participants. In reporting this study, we followed the relevant aspects of the

consolidated criteria for reporting qualitative research (COREQ), [See Supplementary File 3].

This method was chosen as it enabled the identification, analysis and interpretation of patterns and meaning within qualitative data. In addition, this design is not tied to a particular epistemological or theoretical perspective making it flexible and appropriate for this study. The emergent themes were checked against the interview guide and study objectives, resulting in the development of a set of major themes. Co-authors of the study verified the emerging themes and contents [2, 4]

References

1. Etikan I: **Comparison of Convenience Sampling and Purposive Sampling**. *American Journal of Theoretical and Applied Statistics* 2016, **5**(1):1.
2. **Raising Awareness through Public Outreach Campaigns** [https://www.sdgaccountability.org/working-with-informal-processes/raising-awareness-through-public-outreach-campaigns/]
3. Moser A, Korstjens I: **Series: Practical guidance to qualitative research. Part 3: Sampling, data collection and analysis**. *Eur J Gen Pract* 2018, **24**(1):9-18.
4. Johnson JL, Adkins D, Chauvin S: **A Review of the Quality Indicators of Rigor in Qualitative Research**. *Am J Pharm Educ* 2020, **84**(1):7120.
