# Supplementary material for: Investigating public awareness, prevailing attitudes and perceptions towards domestic violence and abuse in the United Kingdom: a qualitative study
Source: BMC Public Health. 2022 Nov 8;22:2042. doi: 10.1186/s12889-022-14426-9 (PMC9643966; doi:10.1186/s12889-022-14426-9)
Supplement: Supplementary file 2 — Additional file 2. Interview Guide - core and probe questions, asked in semi structured interviews that relate to participant’s DVA experiences. The research team developed the interview topic guide after defining the research objectives and reviewing relevant literatures [file 12889_2022_14426_MOESM2_ESM.pdf]

Supplementary File 2

Interview Guide – core and probe questions, asked in semi-structured interviews that relate to participant's DVA experiences

1. What are your views & experiences regarding DVA?

- At what point do such behaviours considered excusable/justifiable?
- Barriers/challenges facing victims?
- Why do you think victims stay in an abusive relationship?

2. In your opinion, what are the contributing factors for DVA?
3. Are you more aware of the prevalence of abuse in the community, since the pandemic & lockdown measures? If yes, how did you find out?
4. Do you know the signs of an individual who has been experiencing DVA?
5. Do you know anyone who has been a victim of any form of abuse (friends/ family/ colleague/ male/ female)?
6. If you were the decision maker, what would you do to tackle DVA?
7. Do you think there is enough public awareness? How could we improve public awareness?
8. Do you think there is enough support services? From whom would you seek that support if you or someone you know was experiencing abuse?
9. Should all frontline workers (including shop workers/delivery drivers) have training on identifying & supporting victims?

10. Your views on police responding to DVA incident?

11. What are your views on teaching about DVA at school? At what age should it start?

12. How about training at work?

13. Would you find it acceptable if your GP/nurse at your GP surgery routinely asked about DVA?

14. How could you support victims not coming forward because of fear/dependents/language barriers etc.?

15. Any other suggestions/recommendations?

16. In your opinion, how should we deal with perpetrators? Offer support?

17. Is there anything that we have not talked about that you would like to tell me or you think that I should know?
