# Supplementary material for: Investigating public awareness, prevailing attitudes and perceptions towards domestic violence and abuse in the United Kingdom: a qualitative study
Source: BMC Public Health. 2022 Nov 8;22:2042. doi: 10.1186/s12889-022-14426-9 (PMC9643966; doi:10.1186/s12889-022-14426-9)
Supplement: Supplementary file 4 — Additional file 4. Preliminary codes and related illustrative quotes. A fuller description of the preliminary codes and quotations from individual participants. [file 12889_2022_14426_MOESM4_ESM.pdf]

Supplementary File 4: Preliminary codes & related illustrative quotes

Preliminary codes	Illustrative quotes
**Prevalence**	➢ *'Everyone knows about domestic abuse; it happens'*<br>➢ *'People talk about bullying and physical abuse and not talk much about control, financial or psychological abuse in the domestic setting; physical harm that we see is tip of the iceberg'*
**Personality**	➢ *'Perpetrators definitely have personality issues' and 'victims mainly are submissive in nature'*<br>➢ *They are good at hiding emotional abuse'*
**Insecure; Emotional detachment; Self-confidence/ Self-esteem**	➢ *'Victims don't come forward as they feel insecure and emotionally withdrawn'*<br>➢ *'Feeling not worthy of themselves'; 'no confidence at all'*
**Dependents/Children; childcare**	➢ *'Need to be with him as my daughter needs a father'; who will look after my children'*
**Lack of awareness**	➢ *'Some victims don't realise they are abused'; even some perpetrators not realise they are abusing'*
**Family/friends**	➢ *'Victims rely on the support of their family and friends'*
**Societal norms; Sociocultural; Religious beliefs**	➢ *It's what happens in every household – so it's normal'*<br>➢ *'Victims not seek help because of social conditioning and religious beliefs'*

	➢ *'Most people turn a blind eye and don't want to involve, especially when not affect themselves'*<br>➢ *'Grown up in an abusive environment so it's normal to get abused'*<br>➢ *'in some cultures men are thought of as leaders and women are expected to follow'*<br>➢ *'Many people in such cultures, become complacent and become submissive, and follow the culture the society has prescribed to them; internalise and becomes normal to be abused'*
**Male dominance**	➢ *'Some cultures males are thought of as superior'*<br>➢ *'Looked down by others especially with male victims, as a man you shouldn't be abused'*
**Poverty; financial difficulties/ Loss of job; Unable to cope; Alcohol abuse/ Substance abuse**	➢ *'People turn to alcohol with financial hitches'*<br>➢ *'Finance has a part to play in abuse'*<br>➢ *'Losing jobs is a trigger factor -when there is no relief in terms of how people can adjust in a stressful situation is a recipe for tensions within the house'*
**Marital/relationship satisfaction; Promiscuity**	➢ *'Lack of satisfaction in the relationship results in having affairs which leads to conflict and abuse'*
**Stress /frustration; No means of letting out; Mental illness; Wants control/Powerless**	➢ *'Why people abuse is partly to accept control'*<br>➢ *'Losing jobs is a trigger factor -when there is no relief in terms of how people can adjust in a stressful situation is a recipe for tensions within the house*<br>➢ *''affects mental health'*

**Affluent society**	➢ *'Abuse happens in wealthy societies too, its often ignored because of power'*
**Isolation; Lockdown restrictions/Indoor mandates**	➢ *'Pandemic definitely increased abuse because of the restrictions'*
**Repeat victimisation**	➢ *'Victim might be aware and they might have access to support in terms of social, medical or criminal; but they may not want to access that support. In repeat victimisation, victim does not want to report'*<br>➢ *'Break the cycle'*
**Stigma/blame; Shame/ embarrassment; Convenient to stay with abuser; Private matter**	➢ *'Frightened that partner may somehow find out that they contacted someone for help'*<br>➢ *'embarrassed that somehow let this happen'*
**Belief abuser will change**	➢ *'Not his fault'*<br>➢ *'believe he will change'*
**Victim educated/higher earner**	➢ *'Some men can't accept if their spouse earns more or better educated causing jealousy and abuse'*
**Not had enough of the abuse**	➢ *'Victims not had enough of the abuse yet! So they don't come forward'; 'until you get to a point enough is enough and until you reach that point, they carry on protecting their abuser'*
**Social media**	➢ *'Media not only should raise public awareness but also ensure outlining the available supportive services'*<br>➢ *Sport celebrities should be encouraged to communicate to their audience, especially target our younger generation;*

	Community and church leaders should play a role in preventing abuse'
**Housing/shelters; Safe space/place; Legal, financial assistance**	➢ *'Most people don't know where to get help or where to go for safety' and 'finance is a big factor in accepting abuse' 'not aware of the legislation'*
**Social support; Social silence**	➢ *'Victims don't want to come forward; we need to break the stigma; having posters in the surgery would make people aware that we are not scared to talk about abuse; holistic, patient centred approach is better'*<br>➢ *'Many people in such cultures, become complacent and become submissive, and follow the culture the society has prescribed to them; internalise and becomes normal to be abused'*
**Abuse disguised/overlooked; Fear/judged; Loneliness; Unknown future**	➢ *'Unwillingness to report by victims can be primarily due to fear of going out of their familiar situation to unknown, in terms of housing and support, or fear that perpetrator might come after them even after they have reported'*<br>➢ *'You might think that you won't find someone better'*<br>➢ *'Fear of unknown, fear of leaving, fear of starting again, fear of admitting to people that it has happened to you'*<br>➢ *'For many women, when they first disclose they are experiencing domestic abuse no one listens to them. No one consults them about how to stop the abuse, despite the fact that no one knows the abuser better than they do'*<br>➢ *'What happens after reporting? victims might end up with perpetrator even after reporting because the supportive*

	services are not available to take the victim away from the situation'
**Lack of anonymity/ Trust in the system; Police/criminal justice service**	➢ *'Need to target areas where the people would trust; People generally tend to believe what they read if information comes from a trusted source like GP surgery or government organisation. By identifying where the teachable moments are and linking the facts, people will become more alert and gain trust''*<br>➢ **'***Victims don't have faith in the police and law'*
**Keyworkers; Schools/ universities/workplace**	➢ *'Missed opportunities'*
**GPs; Time constraints/remote consultations; Screening for early identification**	➢ *'Missed opportunities'*<br>➢ *'Making victims aware, it is easy to get support and not difficult particularly in GP surgeries would be helpful'*<br>➢ *'GPs and nurses are in an ideal opportunity to identify victims; it takes a few minutes to sensitively ask and potentially support a victim'*<br>➢ *'screening will definitely help as it makes it normal for professionals to ask about abuse'*<br>➢ *'HCP should be trained to be desensitised to questions about DVA – practice makes perfect'*<br>➢ *'When we talk about abuse, we are focusing on patients who seek treatment for physical injury; in terms of awareness, we need tools to detect other types of abuse such as psychological abuse. We need more research'*<br>➢ *'Victims will come forward if people show they are willing to listen to their stories'*

**Education and Training**	➢ *'Education and awareness is the key'*<br>➢ *'Raising public awareness and self-awareness is the key in terms of what should be and shouldn't be acceptable'*<br>➢ *'Identify teachable moments when victims are receptive to receiving information; for example, when victims report injury or harm attending the maternity unit, police station'*<br>➢ *'Everyone can play a role in ending domestic abuse; tackling domestic abuse is everyone's business. Together we can create a society that no longer tolerates abuse'*
**Prevention**	➢ *'Tackling domestic abuse to be everyone's business. Together we can create a society that no longer tolerates abuse'*<br>➢ *'Challenge the culture that allows domestic abuse to thrive'*<br>➢ *'Lives are lost', 'prevention and education is how we tackle the problem'*<br>➢ *'Raising public awareness and self-awareness is the key in terms of what should be and shouldn't be acceptable'*<br>➢ *When we talk about abuse, we are focusing on patients who seek treatment for physical injury; in terms of awareness, we need tools to detect other types of abuse such as psychological abuse. We need more research'*
**'Melt the iceberg' of DVA**	➢ *'People talk about bullying and physical abuse and not talk much about control, financial or psychological abuse in the domestic setting; physical harm that we see is tip of the iceberg'*<br>➢ *'They are good at hiding emotional abuse'*

**Abusers needing support; Behavioural change; Counselling; Anger management; Rehabilitation**	➢ *‘Education about how they would feel if they experience abuse, like victims’ and enrolling on awareness training and education could benefit’*<br>➢ *‘Criminalising perpetrators is important, but education is equally important; Criminal justice system needs to be adopted if we want to make a change to the future behaviour of abusers; by building within the frame of sentence, an education such as attending an awareness course to reduce the sentence could help with avoidance of repeat victimisation and repeat cycles’*
**Referral pathways /Self-referral**	➢ *‘Clinicians, as service providers not only need to make people aware that any kind of abuse is unacceptable and not the way anyone should live, but also should outline what support provision is available - no point asking victim to phone the number to report, the question is what type of support is available and what is the logistics’*<br>➢ *‘No point being aware of domestic abuse, people should be clear on the pathway’*<br>➢ *‘Media not only should raise public awareness but also ensure outlining the available supportive services’*<br>➢ *‘Making victims aware, it is easy to get support’*
**Schools; Childhood experiences**	➢ *‘Children may end up being perpetrators or victims in the future, and we need to catch them early; essential to incorporate teaching about domestic abuse in a structured way within the curriculum’*<br>➢ *‘Children learn their behaviour by watching their parents; we need external and internal source to educate children*

	so that it's a united learning that recognises the unacceptable behaviour'<br>➢ *'Education of children aids changing generation attitude - may lessen abuse in the future'*<br>➢ *'Domestic abuse is a learned behaviour, and it should start early on at school, like safe sex and sexual health; school is a safe-haven, children should feel they could talk to teacher, and it is ok to talk'*<br>➢ *'Children are most vulnerable and forgotten at the moment; there seems to be less emphasis on the family unit at present'*<br>➢ *'Should start from early age, as children need to know right from wrong; then you are raising a culture of people who have awareness'*<br>➢ *'Children should know what is acceptable behaviour so that they can identify if they are being abusive themselves, most of the time, abusive behaviour starts with normal behaviour and it becomes a controlled mechanism because they are fearful of something'*

1
