# Supplementary material for: Investigating public awareness, prevailing attitudes and perceptions towards domestic violence and abuse in the United Kingdom: a qualitative study
Source: BMC Public Health. 2022 Nov 8;22:2042. doi: 10.1186/s12889-022-14426-9 (PMC9643966; doi:10.1186/s12889-022-14426-9)
Supplement: Supplementary file 5 — Additional file 5. Preliminary codes, initial thematic framework and final categories and subcategories. A fuller description of the themes and resultant framework. [file 12889_2022_14426_MOESM5_ESM.pdf]

Supplementary File 5: Preliminary codes, initial thematic framework and final categories and subcategories

Preliminary codes

Prevalence
Personality
Insecure
Emotional detachment
Self-confidence/ Self-esteem
Dependents/Children
Lack of awareness
Family/friends
Sociocultural
Religious beliefs
Male dominance
Societal norms
Poverty/financial difficulties
Affluent society
Education
Isolation
Lockdown restrictions/Indoor mandates
Childhood experiences
Repeat victimisation
Social media
Loss of job
Unable to cope
Stress /frustration
No means of letting out
Marital/relationship satisfaction
Promiscuity
Mental illness
Alcohol abuse/Substance abuse
Wants control/Powerless
Victim educated/higher earner
Stigma/blame them
Shame/embarrassment
Convenient to stay with abuser
Private matter
Belief abuser will change
Not had enough of the abuse
Housing/shelters
Safe space/place
Legal, financial assistance
Childcare
Social support
Social silence
Abuse disguised/overlooked
Fear/judged
Unknown future
Loneliness
Lack of anonymity/trust
Police/criminal justice service
Keyworkers
Schools/ universities/workplace GPs
time constraints/remote consultations
Screening for early identification
Training
Prevention
'melt the iceberg' of DVA
Counselling
Abusers needing support
Behavioural change
Anger management
Rehabilitation
Referral pathways /Self-referral

→

Initial thematic framework

1. Experience
2. Personality types
3. Feelings & Beliefs
4. Sociocultural
5. Religious beliefs
6. Circumstances
7. Finance
8. Children
9. Substance abuse
10. Vulnerable/ill-health

---

11. Social conditioning
12. Cultural/religious constraints
13. Circumstances
14. Perception
15. Fear
16. Physical & mental health
17. Education & training
18. Awareness
19. Accessible support
20. Communication
21. Children & other dependents
22. Lack of tools to identify 'hidden abuse'

---

23. Improve public awareness
24. Improve self-awareness & holistic wellbeing
25. Promote social support & break social silence
26. Raise awareness in school curriculum
27. Workforce training
28. Support from family/friends
29. Housing/shelter
30. Financial assistance
31. Legal support
32. Counselling
33. Improved communication
34. Accessible support services
35. Screening/ better tools to help identify 'hidden abuse'

→

Final categories & sub-categories

1-Contributing Factors

- Victims
- Perpetrators

2- Challenges Facing Victims

- Barriers to seeking support

- **Future Interventions**
  - Raising Awareness
  - Education & Training
  - Routine signposting to screening tools for DVA
  - Guidance & mobilization of support Services
  - Sustained funding for targeted interventions
